# Wavefront Correction for Extended Sources Imaging Based on a 97-Element MEMS Deformable Mirror

**DOI:** 10.3390/mi16010050

**Published:** 2024-12-31

**Authors:** Huizhen Yang, Lingzhe Tang, Zhaojun Yan, Peng Chen, Wenjie Yang, Xianshuo Li, Yongqi Ge

**Affiliations:** 1 Engineering School of Networks & Telecommunications, Jinling Institute of Technology, Nanjing 211169, China; yanghz@jit.edu.cn (H.Y.); 2307010003@stu.jit.edu.cn (L.T.); chenpeng375@jit.edu.cn (P.C.); 13151522250@163.com (W.Y.); lxs4219@163.com (X.L.); gyq0406136790@163.com (Y.G.); 2 The Astronomical Optical Instrument Group, Shanghai Astronomical Observatory, Chinese Academy of Sciences, Shanghai 200030, China

**Keywords:** extended objects, wavefront correction, deformable mirror, MEMS, sharpness

## Abstract

Adaptive optics (AO) systems are capable of correcting wavefront aberrations caused by transmission media or defects in optical systems. The deformable mirror (DM) plays a crucial role as a component of the adaptive optics system. In this study, our focus is on analyzing the ability of a 97-element MEMS (Micro-Electro-Mechanical System) DM to correct blurred images of extended sources affected by atmospheric turbulence. The RUN optimizer is employed as the control method to evaluate the correction capability of the DM through simulations and physical experiments. Simulation results demonstrate that within 100 iterations, both the normalized gray variance and Strehl Ratio can converge, leading to an improvement in image quality by approximately 30%. In physics experiments, we observe an increase in normalized gray variance (NGV) from 0.53 to 0.97 and the natural image quality evaluation (NIQE) from 15.35 to 19.73, representing an overall improvement in image quality of about 28%. These findings can offer theoretical and technical support for applying MEMS DMs in correcting imaging issues related to extended sources degraded by wavefront aberrations.

## 1. Introduction

The Adaptive Optics (AO) system [1,2], widely acknowledged as a highly effective technique for enhancing optical imaging, has gained extensive application in various fields in recent years. The fundamental principle of the AO system is to utilize a wavefront corrector to correct aberrant wavefronts and obtain clearer imaging. The imaging correction for extended sources [3,4,5] is an important application of AO technology, which is more complex than traditional point source imaging and often affected by significant optical aberrations. The wavefront corrector plays a crucial role in the AO systems by dynamically adjusting its surface shape to compensate for wavefront aberrations caused by atmospheric turbulence, defects in optical components, or other environmental factors. The phase modulation of the wavefront corrector can be achieved through changes in refractive index or optical path length. By employing a suitable wavefront corrector combined with advanced control algorithms, the AO system can achieve rapid and precise correction capabilities even in dynamic and complex environments, significantly improving the sharpness of extended object imaging.

Commonly used wavefront correctors include different types of DMs (deformable mirrors) and SLMs (spatial light modulators) [6], which operate via distinct mechanisms. Currently available DMs encompass thin film DM [7], piezoelectric DM [8,9,10], separate actuator continuous surface DM [11], splice sub-mirror DM [12], and MEMS (Micro-Electro-Mechanical System) DM among others. These devices find extensive applications in astronomical observations [13], biological microscopies [14], beam shaping in laser processing [15,16], and other fields. The actuator of MEMS DM is actuated by electromagnetism and exhibits a distinctive structure [17]. Previous research has demonstrated that the primary advantage of this DM is its linear control capability. A comparison with conventional piezoelectric and electrostrictive material-based DMs reveals that this particular DM stands out due to its simplicity, environmental friendliness, and potential advantages across various application [18,19,20]. Furthermore, its compatibility with CMOS technology [21] enables improved system response frequency and enhanced accuracy compared to traditional DMs, making it a promising direction for current DM research.

To investigate the correction capability of the MEMS DM in imaging extended objects degraded by wavefront aberrations, we established an AO system comprising a 97-element MEMS DM from Alpao and an image sensor. The imaging correction results under different aberration conditions are analyzed through simulations and physical experiments. The RUNge Kutta optimizer (RUN) [22] is employed as the control algorithm for the AO system [23]. RUN is a metaphor-free, swarm-based metaheuristic algorithm that utilizes the fourth-order Runge Kutta (RK) mathematical approach to calculate gradients and has demonstrated its capability in handling various optimization problems.

The Section 2 provides details on relevant parameters, actuators arrangement and response functions of the DM. Section 3 describes the simulation results for wavefront correction and imaging correction under different turbulence conditions. The Section 4 presents experimental findings, and summarizes the study in the conclusion.

## 2. The 97-Element MEMS DM

### 2.1. Basic Parameters

**Figure 1:**
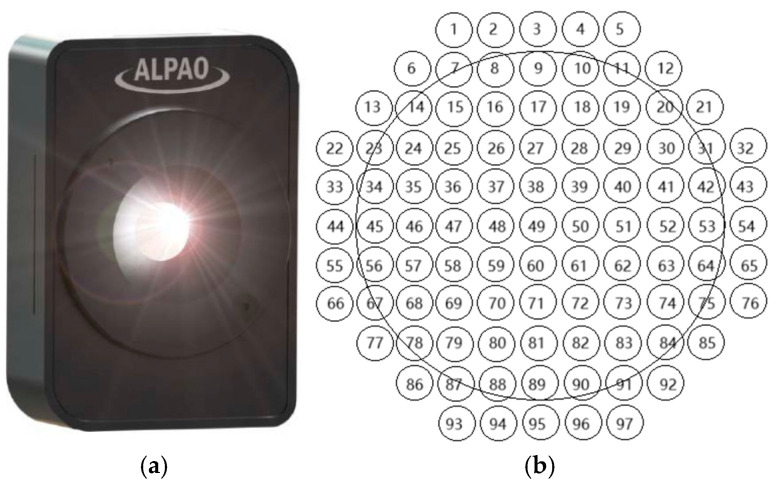
The 97-element MEMS DM, where (**a**) is for physical DM and (**b**) is for spatial distribution of the actuators.

The MEMS deformable mirror is a micro device fabricated from a silicon wafer using semiconductor manufacturing processes. This fabrication process involves sequential layer deposition, culminating in the formation of a multilayer structure, which is subsequently subjected to wet etching. In this paper, we chose a 97-element MEMS deformable mirror manufactured by Alpao as a representative example of an optical component that utilizes MEMS technology specifically designed for precise correction of significant aberrations, such as Zernike high-order aberrations, in order to enhance image quality. Detailed physical characteristics and parameters can be found in Figure 1a and Table 1. The DM, has a full aperture size of 25 mm, with an effective light aperture of 22.5 mm. It is equipped with 97 actuators arranged in a square pattern, spaced at intervals of 2.5 mm between each actuator. Figure 1b illustrates the position arrangement of each actuator, where the circular line denotes the effective light aperture.

### 2.2. Response Functions

**Figure 2:**
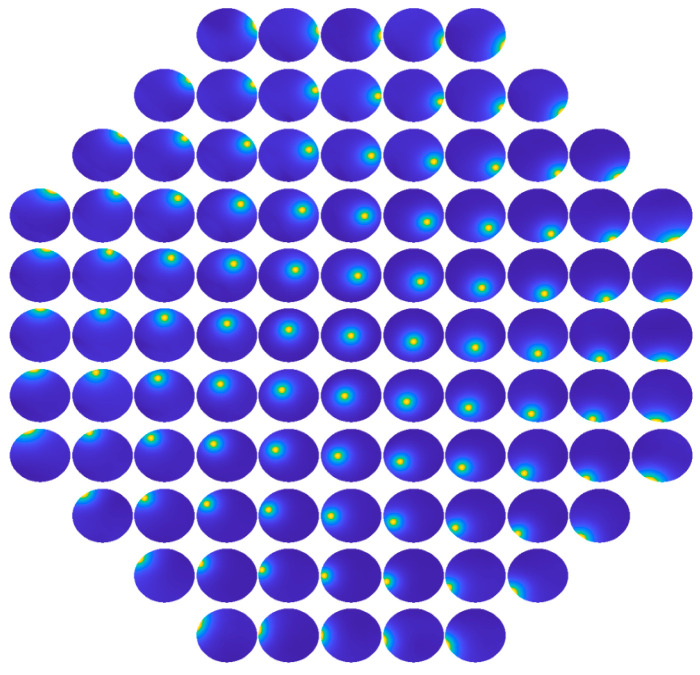
Response functions of 97 actuators.

Each response function can be measured by sequentially applying unit control signals to each actuator. For the 97-element DM used in this paper, we obtained 97 response functions corresponding to its actuators. Figure 2 illustrates the actual 97 response functions of the DM, where each function’s position corresponds to the respective actuator position shown in Figure 1.

**Figure 3:**
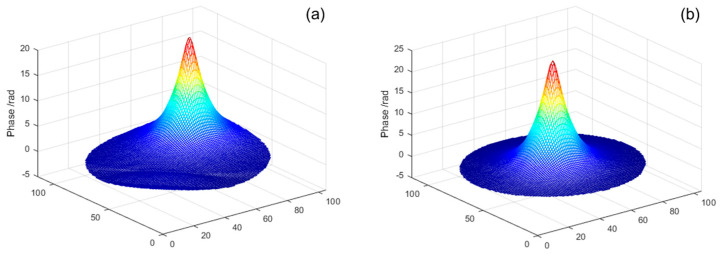

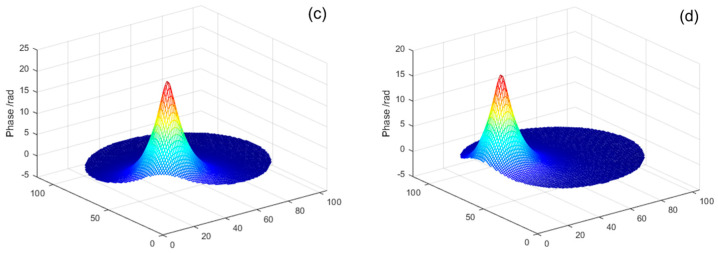
Three-dimensional diagrams of the response functions for several actuators in typical positions, where (**a**–**d**) are for actuator 25, 49, 73, and 81, respectively.

Similarly, Figure 3 displays several three-dimensional shapes of response functions for selected actuators at typical positions: Figure 3a–d correspond to actuator positions 25, 49, 73, and 81 respectively.

## 3. Simulation of Wavefront Correction for Extended Sources Imaging

### 3.1. Wavefront Sensorless AO System Model

**Figure 4:**
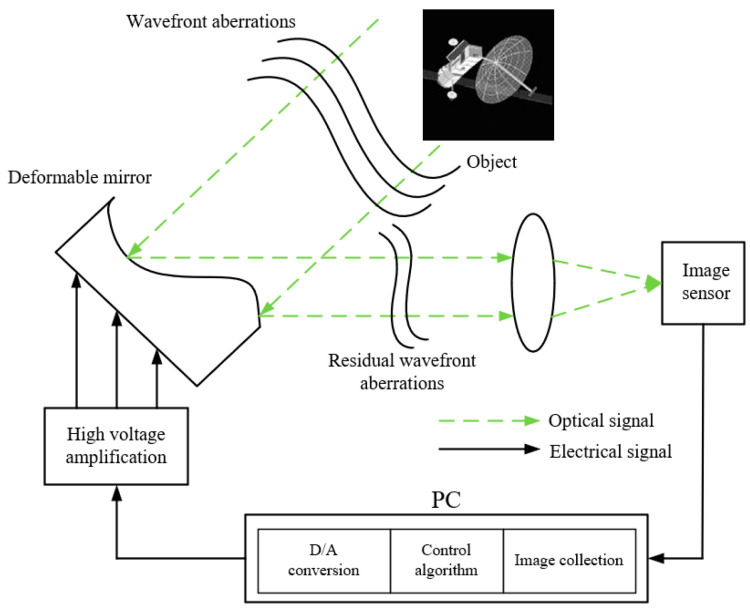
WFS-less AO system model.

The wavefront sensorless AO system, as depicted in Figure 4, mainly consists of a wavefront corrector (a 97-element DM), an image sensor, and a control module integrated into a personal computer.

The aberrant wavefront is reflected by the DM and directed onto the image sensor through the imaging lens. The control module acquires imaging information from the image sensor and generates control signals of DM using specific control method based on imaging information. These control signals are then applied to the DM actuators via a high-voltage amplifier, resulting in the generation of compensation phases. Subsequently, the residual wavefront serves as the input for further closed-loop correction until predetermined conditions are satisfied.

The gray variance function has been identified as the optimal metric function for the control algorithm, based on an analysis of various sharpness functions [24]. This allows for effective high-resolution imaging correction for different extended objects. In this paper, the metric function NGV used in the control algorithm is determined by calculating the ratio between the gray variance of a blurred image and that of an ideal image.

(1) $$NGV=F_{current}/F_{ideal}.$$

The gray variance function F can be expressed as follows.

(2) $$F=\sum_{x}\sum_{y}{\left[f(x,y)-H\right]}^{2},$$

where f(x,y) is the image plane, (x,y) is the image plane coordinates and H is the average of all pixel gray levels. H can be obtained according to Equation (3).

(3) $$H=\frac{1}{MN}\sum_{x}\sum_{y}f(x,y),$$

where M and N are the image sizes. The image gray variance function serves as an evaluation metric for image quality, representing the degree of dispersion in the distribution of pixel gray values. A wider range of gray values in the pixel distribution corresponds to a clearer image. The larger the NGV, the clearer the image, and the maximum NGV is 1. 

Meanwhile, the Strehl Ratio (SR) is used as a metric to evaluate the correction performance of AO system. The SR is defined as follows.

(4) $$SR=\frac{Max(I(u,v))}{Max(I_{0}(u,v))},$$

where I(u,v) is the far-field intensity distribution of wavefront aberrations, and $I_{0}(u,v)$ is that of the ideal plane wavefront. The far-field intensity distribution here is actually the point spread function of the imaging system. A larger SR indicates that the correction capability of the AO system is stronger, and the maximum value of SR is 1.

In addition, considering that the work in this paper involves the imaging correction of extended objects, that is, the correction of blur images affected by atmospheric turbulence to relatively clear images, we also use the Natural Image Quality Evaluator (NIQE) [25], a classical image evaluation standard in the field of image processing, as an evaluation of image sharpness to analyze the imaging correction capability of the wavefront sensorless AO system for extended objects. The formula is as follows:

(5) $$NIQE(v_{1},v_{2},\sum_{1},\sum_{2})=\sqrt{\left({\left(v_{1}-v_{2}\right)}^{T}{\left(\frac{\sum_{1}+\sum_{2}}{2}\right)}^{-1}(v_{1}-v_{2})\right)},$$

where $v_{1}$ is the mean vector of the natural Multivariate Gaussian (MVG) model, $v_{2}$ is the mean vector of the blur image MVG model, $\sum_{1}$ is covariance matrix of natural MVG model and $\sum_{2}$ is covariance matrix of distorted image MVG model. The higher the value of NIQE, the better the image quality.

### 3.2. Simulation Results and Analysis

The wavefront aberrations to be corrected were generated using Roddier’s method [26] as multi-frame phase screens. These phase screens follow the Kolmogorov power spectrum model and exhibit no correlation between them. Zernike polynomials ranging from mode 3 to 104 (excluding the tilt term) were utilized for constructing these phase screens. The turbulence level was quantified by $D/r_{0}$, where D represents the telescope aperture and $r_{0}$ denotes the atmospheric coherence length. The RUN optimizer is chosen as the control method to simulate the correction capability of the 97-element MEMS DM for extended object imaging under different turbulence levels and noise level. Additionally, considering that Gaussian noise is a common type of noise in optical imaging, we acknowledge its presence in this paper.

**Figure 5:**
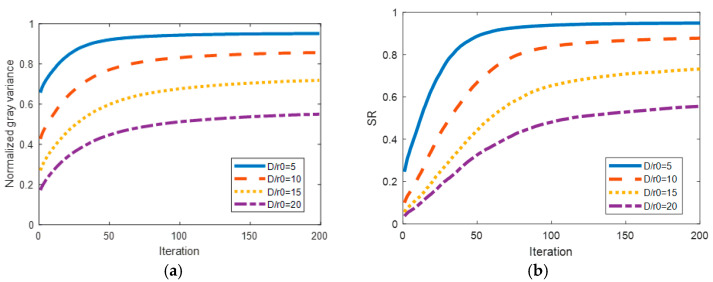
Averaged normalized gray variance and SR curves under four different turbulence levels, where (**a**) is for normalized gray variance and (**b**) is for SR.

The levels of Atmosphere turbulence $D/r_{0}$ are set at 5, 10, 15, 20 respectively. For each turbulence level, a total of 200 phase screens are randomly selected as wavefront aberrations to be corrected. The averaged normalized gray variance NGV is then calculated as the correction result for each turbulence level. Additionally, the changes in far-field intensity, which represent the variations in SR, were also recorded to investigate the correction results of wavefront aberrations. The signal-to-noise ratio (SNR) is set at 10 dB. Figure 5 illustrates the curves depicting the averaged normalized gray variance NGV (a) and SR (b).

The wavefront sensorless AO system with the 97-element DM and the RUN optimizer demonstrates convergence under four different turbulence levels, as depicted in Figure 5. It is observed that higher turbulence levels lead to increased wavefront aberration and slower convergence rates. However, even at a turbulence level of D/r_0_ = 20, the system only requires approximately 100 iterations to achieve convergence.

**Figure 6:**
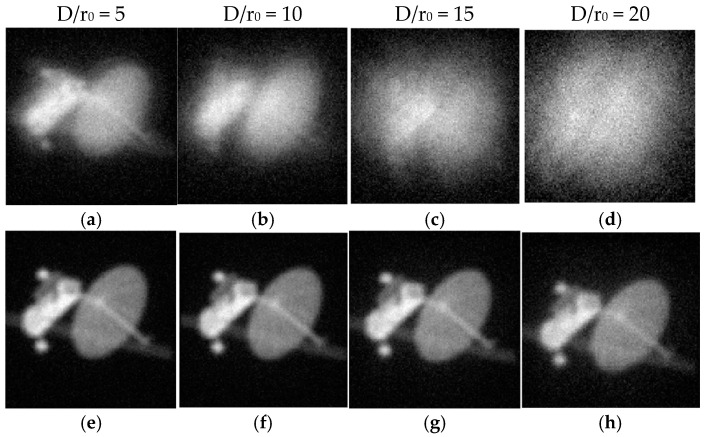
Imaging results before and after correction under different turbulence levels by a single phase screen when the SNR is 10, where (**a**–**d**) are for images before correction and (**e**–**h**) are images after correction.

In order to directly observe the imaging effect of the extended object before and after correction, Figure 6 presents imaging results of a single phase screen under four different turbulence conditions, where Figure 6a–d represent the imaging before correction, and Figure 6e–h represent the imaging after aberration correction. It can be seen from Figure 6 that corrected images exhibit enhanced clarity compared to their pre-correction counterparts. Notably, Gaussian noise with an SNR of 10 was introduced into the imaging system. Although AO technology is capable of correcting wavefront aberrations, it cannot eliminate noise; thus, its presence is evident in the corrected images. However, this also serves as evidence that noise has minimal impact on the correction process, thereby highlighting the anti-noise capability of this AO system.

**Figure 7:**
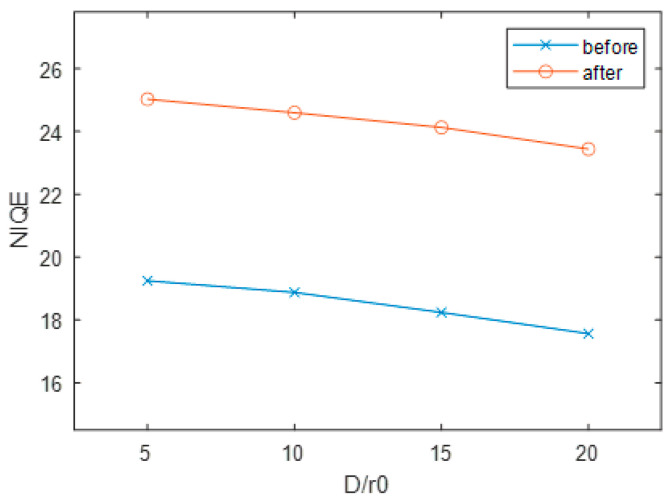
The image quality before and after correction.

**Figure 8:**
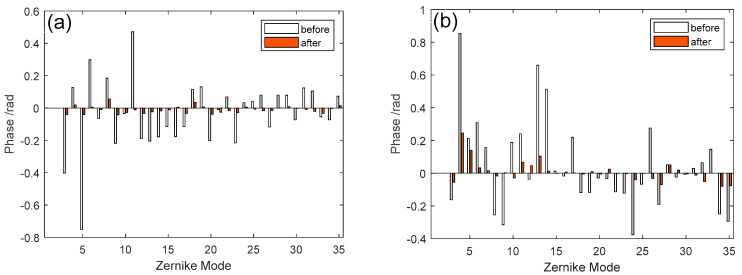

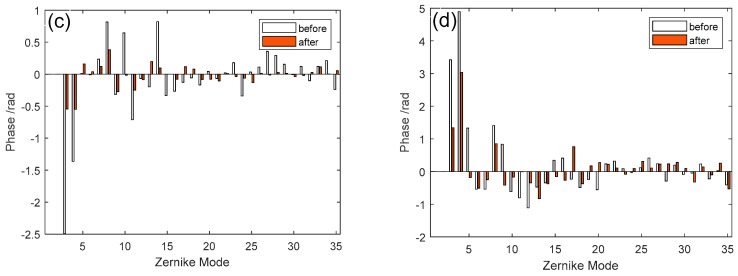
Comparison of Zernike coefficients 3–104 before correction and after correction, where (**a**) is for D/r_0_ = 5, (**b**) is for D/r_0_ = 10, (**c**) is for D/r_0_ = 15, and (**d**) is for D/r_0_ = 20.

Then quantitative method is used to analyze the effect of the extended object imaging correction. The Natural Image Quality Evaluator NIQE before and after imaging correction under four different turbulence conditions was calculated according to Equation (5) in Section 3.1, and the results are presented in Figure 7. It can be observed from Figure 8 that the values of NIQE have significantly improved after correction compared to those before correction, with an increase of more than 30% in imaging effects under all four turbulence conditions. Particularly, when the turbulence level is high, the metric increases from 17.56 to 23.44. This demonstrates that even under strong turbulence levels, the utilized DM exhibits a robust correction capability.

**Figure 9:**
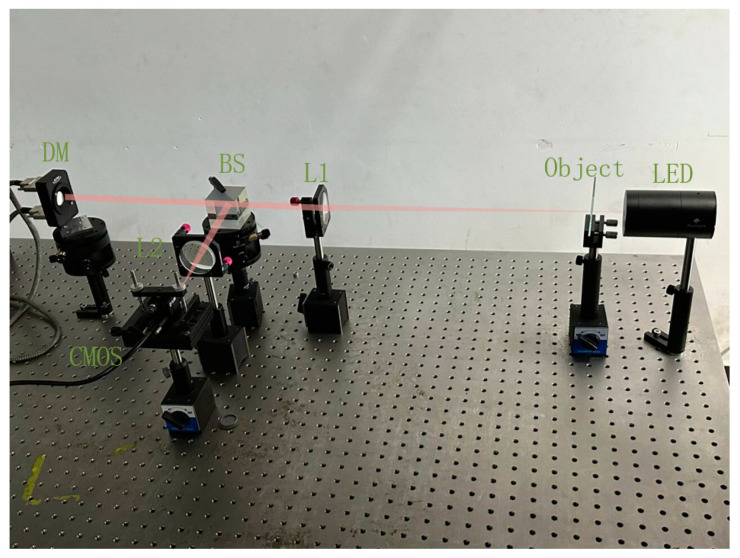
Physical diagram of the experimental system.

The Zernike coefficients of the wavefront aberrations corresponding to Figure 8 before and after correction are shown in Figure 9, where (a) is for D/r_0_ = 5, (b) is for D/r_0_ = 10, (c) is for D/r_0_ = 15, and (d) is for D/r_0_ = 20. Since low-order Zernike modes account for a large amount, we present Zernike coefficients ranging from 3 to 35 order.

It can be seen that after correction, the Zernike coefficient of each order is significantly lower than that before correction, which proves the 97-element MEMS DM has good correction ability for extended object imaging.

## 4. Physical Experiments and Results Analysis

**Figure 10:**
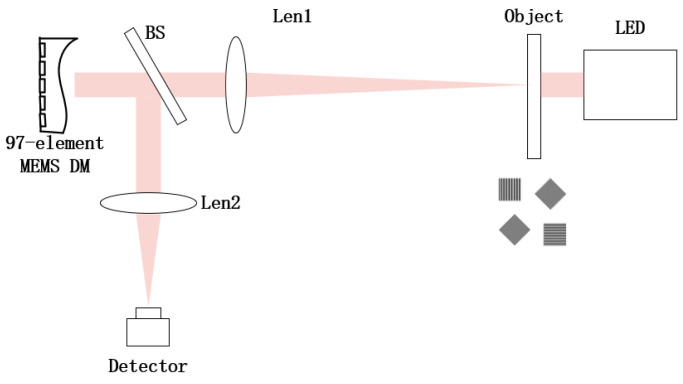
Schematic diagram of the experimental system.

The experimental setup is illustrated in Figure 9 and its corresponding schematic diagram is shown in Figure 10. The light source is positioned in close proximity to the resolution plate, which is situated at the focal point of lens L1. The resolution board is used as the extended source to be corrected. The beam traverses through the resolution plate, passes through lens L1, and splits into parallel beams via a beam splitter. Subsequently, the reflected beam from the DM returns to the splitter mirror, passes through lens L2, and finally reaches the image sensor located at focal point of L2.

The light source utilized was an LED with a central wavelength of 570 nm. The extended object is a resolution test board, model GH-YP832, consisting of 25 sets of stripes in four directions, featuring line widths ranging from 2.5 μm to 500 μm. The 97-element MEMS DM produced by Alpao was used to correct wavefront aberrations. The image sensor is a 16-bit CMOS camera, model Blackfly S BFS-U3-23S3M, produced by FLIR. The focal length of lens L1 and L2 are 400 mm and 100 mm respectively, which form a 4-F system.

The optical system’s parameters facilitate the calculation of theoretical diffraction limit. The theoretical diffraction limit can be determined using the formula 1.22λf/d with given parameters as follows. Given the values of central wavelength (λ = 570 nm), the focal length (f2 = 100 mm) and the effective aperture (d = 22.5 mm), the diffraction limit of the experimental system is 24.73 μm. It shows that we can see lines wider than 24.73 μm. Considering that the resolution board is a light and dark stripe, the width to distinguish a pair of adjacent light and dark stripes should be 49.5 μm.

### Experimental Results and Analysis

**Figure 11:**
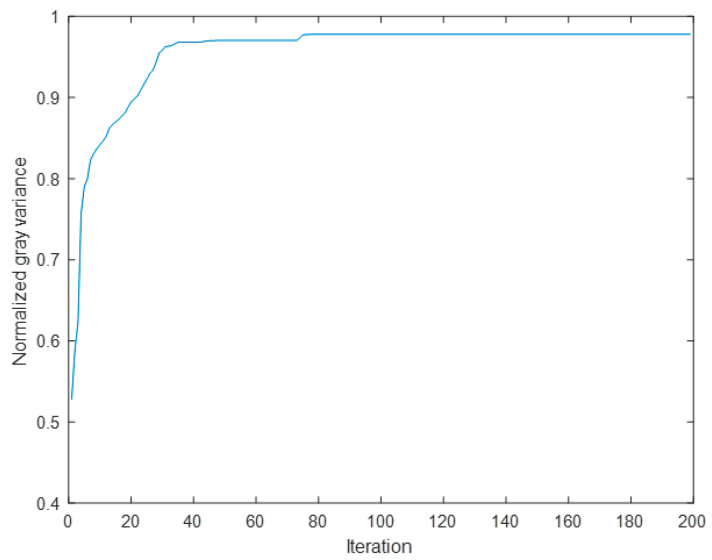
The convergence curve of the normalized gray variance.

The variation curve of normalized gray variance NGV is presented in Figure 11. Upon careful examination of Figure 11, it becomes evident that the wavefront sensorless AO system equipped with a 97-element DM achieved convergence after 30 iterations, resulting in a significant improvement in imaging quality as indicated by the change in value from 0.53 to 0.97.

Next, we use the Equation (5) to calculate the natural image quality evaluator NIQE of the corrected image. Comparison of image sharpness before and after correction is given in Table 2. From Table 2, we can see the value of NIQE increased from 15.35 before correction to 19.73 after correction, representing a 28.6% improvement in image quality.

**Figure 12:**
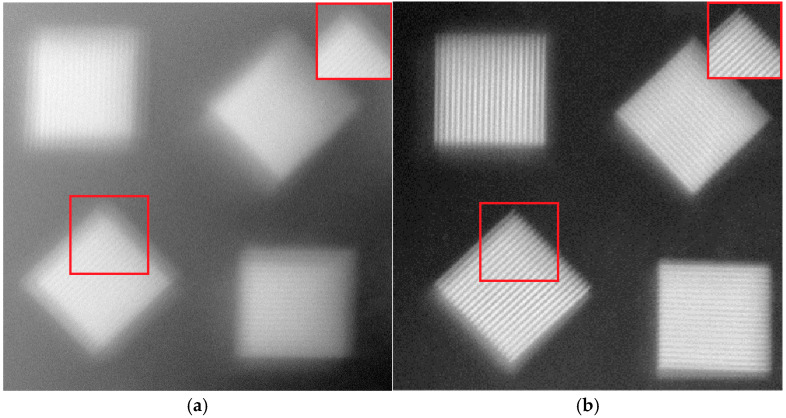
The imaging comparison before and after correction, where (**a**) is before correction and (**b**) is after correction.

The imaging comparison before and after correction is shown in Figure 12. It can be seen from Figure 12 that the fringes before correction are very fuzzy, while the fringes after correction are clearly visible, and the imaging sharpness is greatly improved. There are 20 clear lines on the corrected resolution board and the width to distinguish a pair of adjacent light and dark stripes is 50 μm, which is very close to the diffraction limit. The red frame in the image is locally enlarged for comparison.

## 5. Conclusions

The imaging correction for extended sources is an important application of AO technology, which is more complex than traditional point source imaging. The wavefront corrector plays a crucial role in the AO systems by dynamically adjusting its surface shape. To assess the correction capability of the MEMS DM in imaging extended objects degraded by wavefront aberrations, we established an AO system comprising a 97-element MEMS DM from Alpao and an image sensor. The RUN optimizer is employed as the control method to evaluate the correction capability of the DM through simulations and experiments.

The results of extended object imaging correction under different turbulence levels and noise conditions demonstrate that the AO system can converge quickly and has strong anti-noise ability. The normalized gray variance and Strehl Ratio can converge within 100 iterations, leading to a significant improvement in image quality by approximately 30%. In physics experiments, the normalized gray variance increases from 0.53 to 0.97, and the natural image quality evaluator increases from 15.35 to 19.74, representing an improvement in image quality of around 28%. The fringes before correction appear blurry, whereas after correction they become clearly visible with greatly improved imaging clarity. Notably, there are 20 clear lines on the corrected resolution board, which is very close to the diffraction limit of AO imaging system. Above results prove that the wavefront sensorless adaptive optical system based on MEMS deformable mirror has the ability of image correction for extended objects. These research findings provide theoretical and technical support for applying MEMS DMs in imaging corrections for extended sources affected by wavefront aberrations. For example, In astronomy, MEMS DM can be integrated into ground-based telescopes to correct the optical wavefront distortion caused by atmospheric turbulence in real time and improve the resolution of astronomical observations. In ophthalmology, MEMS DM can be applied in ophthalmic imaging devices to improve the clarity of retinal imaging and help doctors diagnose eye diseases more accurately, and so on. We hope to provide reliable data for subsequent researchers.

## Figures and Tables

**Table 1:** Performance parameters of the 97-element MEMS DM.

Model	Drives Number	Aperture Diameter (mm)	Drive Spacing (mm)	Radial Drives Number	Defocusing /Astigmatic (um)	Stable Time (ms)	Size (mm)
DM97-25	97	22.5	2.5	11	30	1.5	62 × 84 × 23

**Table 2:** Comparison of image sharpness before and after correction.

	NGV		NIQE
Before correction	0.53		15.35
After correction	0.97		19.73
Percentage increase			28.6%

## Data Availability

Data underlying the results presented in this paper are not publicly available at this time but may be obtained from the authors upon reasonable request.
